# HoloSelecta dataset: 10’035 GTIN-labelled product instances in vending machines for object detection of packaged products in retail environments

**DOI:** 10.1016/j.dib.2020.106280

**Published:** 2020-09-08

**Authors:** K. Fuchs, T. Grundmann, M. Haldimann, E. Fleisch

**Affiliations:** a ETH Zurich, Switzerland; b D ONE, Switzerland

**Keywords:** Computer vision, Deep learning, Object detection, Packaged products, GTIN

## Abstract

To assess the potential of current neural network architectures to reliably identify packaged products within a retail environment, we created an open-source dataset of 295 shelf images of vending machines with 10’035 labelled instances of 109 products. The dataset contains photos of vending machines by the provider Selecta, the largest European operator of vending machines. The vending machines are a mix of machines in public and private office spaces. The vending machines contain food as well as beverage products. The product instances in the vending machine images are labelled with bounding boxes, where a bounding box encapsulates the entire product with as little overlap as possible. The labels corresponding to the bounding box consist of a structured, human-readable labels including brand, product name and size as well as the GTIN of the product. The GTIN is the global standard to identify products in the retail environment and therefore increases the value as a dataset for the retail industry. Contrary to typical object detection datasets that choose labels at a higher level such as a can or bottle for a much wider variety of objects, this dataset chooses a far more detailed label that depends less on the shape but rather on the exact design of the product. The dataset falls into the category of object detection datasets with a large number of objects, which next to the GTIN label, represents a main differentiator of the dataset to other object detection datasets.

## Specifications Table

**Table:** 

Subject	Artificial Intelligence
Specific subject area	Labelled dataset for object detection of packaged products in retail environments, a subfield of computer vision and machine learning.
Type of data	Table Image
How data were acquired	The images were acquired with cameras (professional and mobile) by the authors. The images of the vending machine were taken with the consent of the vending machine company. The labels for the products were generated with the open source labelling tool labelImg [1], in the PascalVOC format. Drawing bounding boxes were manually added in the tool.
Data format	Raw
Parameters for data collection	Vending machines were photographed in different physical locations under different light conditions: indoor vs. outdoor, daytime vs. evening, and with different assortments. The data collection lasted several weeks to ensure that products were withdrawn & refilled from the vending machine, as required for the related studies.
Description of data collection	The images were taken with cameras (professional and mobile) by the authors who took the pictures of the vending machine.
Data source location	City/Town/Region: Zurich Country: Switzerland Position of vending machines near main trains station in Zurich, Switzerland: the exact positions for publicly available machines can be found in the ‘Selecta find & pay app’ by the Selecta AG available in the Swiss Android Store.
Data accessibility	Repository name: HoloSelecta Dataset Mendeley Data Direct URL to data: https://data.mendeley.com/datasets/gz39ggf35n/1
Related research article	Future Generation Computer System: Supporting Food Choices in the Internet of People: Automatic Detection of Diet-related Activities and Display of Real-time Interventions via Mixed Reality Headsets [2]

## Value of the Data

Missing publicly available, labelled image data of packaged products is one of the most pressing limitations in research on product detection and identification in retail environments. Therefore, this dataset is one of very few publicly available image datasets (Table 3), labelled at the product level in densely packed scenes (i.e. vending machines).

We maintain a list of relevant existing public datasets (Table 1) on packaged products in retail environments on Github: Link.

• Unlike existing datasets, the HoloSelecta dataset is labelled with global trade item numbers (GTIN), which allows for data fusion with product master data. Thanks to the GTIN-annotation, multiple research avenues could be supported by this dataset via data fusion with nutritional composition (e.g. for health applications), logistics data (e.g. for checking shelf compliance) or prices (e.g. for self-checkout).
• Moreover, this dataset contains edge cases that are hard to identify (e.g. reflections, backside-oriented products, etc.). Such important corner cases of object detection scenarios occur in the real world and are therefore important to be considered. The edge cases are not labelled explicitly but such labels will reflect realistic conditions and likely end up with lower accuracy in an object classification pipeline.
• In addition, this dataset contains product meta data such as prices, brands and nutritional composition of products sold in the vending machine. This data can hence be used to guide shoppers towards an affordable or a rather healthy product, for example within a mixed reality setup where a user uses a smartphone or headset to view the products.
• The data can be used to study object detection performance per se in densely packed or retail contexts using typical object detection metrics. Further the data can be used to power user studies that look at the effect of such technology on user behavior.

## 1 Data Description

The dataset consists of image and label file pairs, where the name of the file is the same (i.e IMG_20190206_175724) and the difference between the image and the label file is the ending. Thus, there are 295 image files and 295 label files. Images end with .jpg or .png and label files end with .xml. The image files are the raw images, all images are upright and uncropped, and they come in differing resolutions. The label files use the widely used PascalVOC standard to label products on the corresponding image. The PascalVOC format uses and XML structure (Table 2), which look as follows (only relevant attributes are given other attributes are results of storage and processing or simply unused and without value):

**Table 1:** Overview over existing publicly available labelled image datasets of packaged products in retail environments.

Name	# Classes	# Instances	# Images	GTIN	Product data	Citation
Holoselecta	109	10035	295	Yes	Yes, e.g. nutrients/price/brand	[2]
Grozi-3.2K	3235	3235	3235 + 680	No	No	[3]
Grozi120	120	120	720 + 4973	No	No	[4]
SKU110K	110,712	∼1.74 * 10^6	11,762	No	No	[5]

**Table 2:** HoloSelecta image annotation follows the established PascalVOC specification.

<annotation>
<filename>IMG_20181218_175304.jpg</filename>
<size>
<width>3480</width>
<height>4640</height>
<depth>3</depth>
</size>
<object>
<name>jacklinks_beefjerkyorginal__25__4047751730219</name>
<bndbox>
<xmin>527</xmin>
<ymin>54</ymin>
<xmax>1006</xmax>
<ymax>569</ymax>
</bndbox>
</object>
…
</annotation>

The label file states the corresponding image file as filename, the size of the corresponding image (width, height, number of channels) and all objects on the image. The object class is repeated once per object on the image. For each object the name states the label of the product. The label consists of six parts which are separates by a single _ (double __ mean the value at the position between is “”). Positions 1 to 5 are a human readable expression of the GTIN which resides at position 6. The first element is the brand (i.e fanta), the second position states the product specialization (i.e. zero), the third states the shape (i.e can vs bottle), the fourth states the size (for drinks 33 = 33cl, for snacks 25 = 25 g) and the fifth positions states if the product is a multipack of the product (i.e package of 6 bottles). The object further carries the position of the bounding box as ‘bndbox’ where ‘x’ corresponds width from left (min) to right (max) and y corresponds to height from top (min) to bottom (max) of the bounding box.

In addition, product meta data is provided in the attached product_meta_data.py. Both datasets, the labelled images and product meta data are linked via the product identifier (e.g. jacklinks_beefjerkyorginal__25__4047751730219 in this example below, Table 3).

**Table 3:** Product data is linked via canonical product name and extendedable via gtin as secondary key (product_meta_data.py).

"jacklinks_beefjerkyorginal__25__4047751730219":{
"gtin"="4047751730219",
"producer"="Jack Link\'s",
"price":3.5,
"price_unit":"CHF",
"weight":25.0,
"weight_unit":"g",
"energy":"289.9032",
"sugar":"14.0",
"sat_fat":"1.0",
"natrium":"1.8999999714648623",
"protein":"46.0",
"fiber":"0.0",
"health_percentage":"0",
}

**Codelist (product data dictionary produced by product_meta_data.py):**

name: Name of a product
gtin: Global trade item number
producer: Organization or brand that produced the product
price and price_unit: Price of product at the vending machine in Swiss francs
weight and weight_unit: Quantity of product in ml (beverages or water) or g (food)
energy: calories (kcal per 100 g or ml of product)
sugar: total sugars (g per 100 g or ml of product)
sat_fat: saturated fatty acids (g per 100 g or ml of product)
natrium: sodium (g per 100 g or ml of product)
protein: protein (g per 100 g or ml of product)
fiber: dietary fiber (g per 100 g or ml of product)
health_percentage: Share of product that is composed of fruit or vegetable or nuts (according to Nutri-Score framework, from 0 to 1 (=100%)
score: FSA points in the Nutri-Score framework from -15 (healthiest) to 40 (unhealthiest)
nutriscore: Nutri-Score letter from A (healthiest) to E (unhealthiest)

## 2 Experimental Design, Materials and Methods

The HoloSelecta dataset was created by selecting a globally representative vending machine setting. We describe this setting in our publication of the HoloSelecta studies [2]. We purchased each product to collect multiple pictures of each item from outside of the vending machine. The aim was to take multiple pictures at varying angles ideally from the front of the product. The idea was to proxy the ‘as-is’ view of a consumer approaching the vending machine, where certain products (especially bottles) may not always appear with their frontal package display.

The dataset was collected to validate the potential of different neural network architectures to detect and identify products within a vending machine. The results in our manuscript indicate promising potential, as accuracy rates yield acceptable results. The dataset consisted of 295 vending machine images in which 10’035 product instances were labelled with *labelImg* [1], an open-source tool for image labelling for computer vision applications (https://github.com/tzutalin/labelImg).

## Ethics Statement

The data collection did not involve any animals or humans (except for the authors who took and labelled the pictures). Therefore, no animals or humans were harmed in the data collection. The dataset does not contain any personal information and therefore does not require ethical approval by our university's ethics commission. Table 1

## Declaration of Competing Interest

The authors declare that they have no known competing financial interests or personal relationships which have, or could be perceived to have, influenced the work reported in this article.
